# Diagnostic accuracy of transcranial Doppler for cerebral vasospasm in aneurysmal subarachnoid hemorrhage: a systematic review and meta-analysis

**DOI:** 10.1186/s13054-026-05849-6

**Published:** 2026-02-02

**Authors:** Yoshihisa Fujimoto, Kohei Yamada, Takuo Yoshida

**Affiliations:** 1 Department of Emergency and Critical Care Medicine, St. Marianna University School of Medicine, Kawasaki, Kanagawa Japan; 2 Department of Traumatology and Critical Care Medicine, National Defense Medical College Hospital, Saitama, 359-0042 Japan; 3 Division of Intensive Care, Department of Emergency and Disaster Medicine, Kashiwa Hospital, The Jikei University School of Medicine, Minato-kuc, 105-8471 Japan

**Keywords:** Aneurysmal subarachnoid hemorrhage, Cerebral vasospasm, Delayed cerebral ischemia, Transcranial Doppler, Diagnostic accuracy

## Abstract

**Background:**

With the increasing adoption of point-of-care ultrasound in neurocritical care, transcranial Doppler (TCD) has gained renewed attention as a bedside monitoring tool for detecting cerebral vasospasm and delayed cerebral ischemia (DCI) in patients with aneurysmal subarachnoid hemorrhage (aSAH). Among cerebral vessels, the middle cerebral artery (MCA) is the most frequently evaluated using point-of-care ultrasound. This study aimed to determine the diagnostic accuracy of TCD for detecting cerebral vasospasm that may lead to DCI in the MCA of patients with aSAH.

**Methods:**

We conducted a systematic literature search of MEDLINE, the Cochrane Central Register of Controlled Trials, Embase, ClinicalTrial.gov, and the WHO International Clinical Trials Registry Platform through December 16, 2025, and manually searched the reference lists of relevant articles. We followed the Preferred Reporting Items for Systematic Reviews and Meta-Analyses guidelines and assessed study quality using the Quality Assessment of Diagnostic Accuracy Studies-2 tool. A meta-analysis was conducted to pool the diagnostic accuracy of TCD for detecting cerebral vasospasm in aSAH. The study protocol was prospectively registered in PROSPERO (CRD42024542692).

**Results:**

Among the 3100 studies identified, 80 underwent full-text review, and 32 studies including 3594 patients were included in the meta-analysis. Pooled sensitivity and specificity were 76% (95% confidence interval [CI]: 70–81%) and 77% (95% CI, 68–84%), respectively, for cerebral vasospasm. The area under the receiver operating characteristic curve was 0.82. The positive likelihood ratio was 3.4 (95% CI, 2.8–4.7), and the negative likelihood ratio was 0.3 (95% CI, 0.24–0.40). Subgroup analyses showed sensitivity and specificity of 76% (95% CI, 69–81%) and 75% (95% CI, 66–83%) using blood-flow velocity in the MCA (28 studies; *n* = 3161), 82% (95% CI, 58–94%) and 88% (95% CI, 64–97%) using the Lindegaard ratio (7 studies; *n* = 1162), and 79% (95% CI, 72–85%) and 82% (95% CI, 66–91%) in studies with low risk of bias (9 studies; *n* = 1223).

**Conclusions:**

TCD demonstrates promising diagnostic accuracy for detecting cerebral vasospasm that may lead to DCI. Incorporating TCD into multimodal neuromonitoring may improve the clinical management of aSAH.

**Supplementary Information:**

The online version contains supplementary material available at 10.1186/s13054-026-05849-6.

## Introduction

Aneurysmal subarachnoid hemorrhage (aSAH) remains a severe condition associated with poor outcomes. Among its major complications, cerebral vasospasm and delayed cerebral ischemia (DCI) are key determinants of poor neurological outcomes [1], prolonged hospitalization [2], and increased healthcare costs [2]. Early recognition of these complications is therefore essential to optimize patient management.

Transcranial Doppler (TCD) ultrasonography has long been used as a noninvasive bedside technique for repeated assessment of cerebral perfusion and for detecting vasospasm, a primary cause of DCI [3]. In contrast to invasive and single-time-point modalities such as cerebral angiography, TCD provides dynamic and continuous evaluation without exposure to radiation or contrast agents [4]. Several studies have reported that TCD demonstrated high sensitivity and specificity for detecting vasospasm and predicting DCI [5, 6]. Accordingly, major clinical practice guidelines endorsed TCD as a noninvasive monitoring tool for patients with aSAH [7, 8]. Recently, with the increasing adoption of point-of-care ultrasound (POCUS) in neurocritical care, the role of TCD as a bedside tool for monitoring vasospasm and DCI has drawn renewed attention in patients with aSAH [9, 10]. Although several systematic reviews have assessed the diagnostic accuracy of TCD for vasospasm and DCI in this population [11, 12], these reviews may not fully reflect the current dissemination of POCUS practice or incorporate the most recent evidence. Moreover, while existing systematic reviews have assessed diagnostic accuracy by combining data from multiple vessels [11–13], it is clinically important to evaluate the middle cerebral artery (MCA) specifically, as it is the most frequently assessed vessel in POCUS.

In this study, we conducted a systematic review and meta-analysis to evaluate the diagnostic accuracy of TCD for detecting cerebral vasospasm that may lead to DCI in the MCA of patients with aSAH.

## Methods

We adhered to the Cochrane Handbook for Diagnostic Test Accuracy [14] and reported our findings according to the Preferred Reporting Items for Systematic Reviews and Meta-Analyses of Diagnostic Test Accuracy Studies (PRISMA-DTA) guidelines [15, 16]. The study protocol was prospectively registered in PROSPERO (CRD42024542692). In this study, the target population was defined as adult patients with aSAH. The index test of interest was TCD for assessing cerebral vasospasm. Thresholds for suspected cerebral vasospasm was based on mean blood flow velocity (MFV) in the MCA or the Lindegaard ratio, calculated by dividing the MCA flow velocity by the flow velocity in the ipsilateral internal carotid artery [17], as specified in each study. For the purposes of this review, we considered as reference standards either angiographic confirmation of vasospasm or a clinical diagnosis of cerebral vasospasm based on neurological examination and neuroimaging (cerebral angiography, computed tomography, or Magnetic Resonance Imaging), as defined in each study.

### Data sources and searches

We conducted a systematic literature search of the Medical Literature Analysis and Retrieval System Online (MEDLINE) via PubMed, the Cochrane Central Register of Controlled Trials (CENTRAL), and Embase from database inceptions through December 16, 2025, and manually searched the reference lists of relevant articles. In addition, we searched the following preregistration sites: ClinicalTrials.gov and the WHO International Clinical Trials Registry Platform (ICTRP). The searches involved combinations of free-text terms and Medical Subject Headings using permutations of the terms “subarachnoid hemorrhage,” “ultrasonography, Doppler, transcranial,” “neurosono,” “echoencephalograph,” “Doppler,” “neurovascular,” “TCD,” and “TCCS” (Additional file 1: Table S1). Methodological search filters were avoided. We also included abstracts presented at national and international conferences, provided that they were subsequently published in journal supplements.

### Study eligibility

We included prospective, retrospective, and observational (cohort or cross-sectional) studies, as well as secondary analyses of randomized controlled trial data, restricted to adult human populations (≥ 18 years). Only English-language publications were included. The publication period was not restricted. We excluded diagnostic case-control studies (two-gate studies) and case studies that lacked diagnostic test accuracy (DTA) data, including true-positive (TP), false-positive (FP), true-negative (TN), and false-negative (FN) values. In addition, studies in which the diagnosis date according to the reference standard was clearly distinct from the date of TCD implementation were considered likely to be aimed at predicting the future onset of DCI rather than diagnosing current cerebral vasospasm, and were therefore excluded.

### Study selection and data extraction

Study selection, exclusion, and data extraction were conducted in a blinded manner and independently checked by two researchers (YF, KY) based on the titles and abstracts. If eligibility could not be determined from the title or abstract, the full text was retrieved. Potentially relevant studies, identified by at least one reviewer, were retrieved and evaluated in full-text form. Any disagreements between the two reviewers were discussed and resolved; if a consensus was not achieved, a third reviewer (TY) acted as an arbiter. The following data were extracted using a predefined data extraction form: study characteristics (author, year of publication, country, design, sample size, clinical settings, conflicts of interest, and funding source); patient characteristics (inclusion and exclusion criteria and clinical and demographic characteristics); index test information (timing of testing, ultrasound protocol, diagnostic cutoffs, and personnel performing the test); reference standard information (data used for diagnosis, definition of DCI, timing of diagnosis, and personnel performing the diagnosis); and diagnostic accuracy parameters (TP, FP, TN, and FN). If the original manuscript did not contain sufficient diagnostic accuracy data, we contacted the authors to request additional information or incorporated available data from prior systematic reviews.

### Quality assessment

We assessed study quality using the Quality Assessment of Diagnostic Accuracy Studies-2 tool (QUADAS-2) [18], which includes four risk-of-bias domains (patient selection, index test, reference standard, and flow and timing) and three domains of applicability (patient selection, index test, and reference standard).

### Data synthesis and statistical analysis

A meta-analysis was conducted to pool the diagnostic accuracy of TCD for detecting cerebral vasospasm. Sensitivity and specificity estimated from individual index studies, along with their respective 95% confidence intervals (CIs), were displayed in forest plots to assess heterogeneity. Synthesis analyses were conducted using Reitsma’s bivariate random-effects model [19] to estimate pooled sensitivities, specificities, positive likelihood ratios, and negative likelihood ratios, accounting for potential heterogeneity among the included studies. Summary estimates were assessed, and their inconsistencies (*I*^*2*^) were calculated to quantify the proportion of total variation across studies attributable to heterogeneity rather than random error. Variability in diagnostic accuracy of TCD for identifying cerebral vasospasm was visually assessed by generating summary receiver operating characteristic (SROC) curves [20] based on estimates from the bivariate random-effects model. The areas under the curves (AUCs) of the SROC curves were presented as summary indicators of predictive accuracy [21]. For statistical inference, restricted maximum likelihood estimation was applied within Reitsma’s model, and the bootstrap method was employed to compute 95% CIs for the AUC. We performed subgroup analysis if the following data were available: the TCD threshold for cerebral vasospasm, diagnostic criteria in the reference standard, and the severity grade of aSAH. Given the uncertainty regarding the optimal diagnostic threshold, all eligible studies were included in the primary meta-analysis to avoid excluding potentially informative evidence solely because of heterogeneity in threshold selection. Sensitivity analyses were also performed using subgroups that excluded studies at high risk of bias, as well as subgroups stratified according to differences in study design characteristics, where such differences were present. Given the absence of evidence for publication bias in DTA studies and the lack of reliable methods for its assessment [22, 23], publication bias was not statistically evaluated. All statistical analyses were conducted using a two-sided alpha error of 5%. All analyses were performed using R version 4.1.2 (The R Foundation for Statistical Computing, Vienna, Austria). Heterogeneity was assessed using the *I*² statistical method, with *I²* > 50% or p-value < 0.05 indicating significant heterogeneity.

## Results

**Fig. 1:**
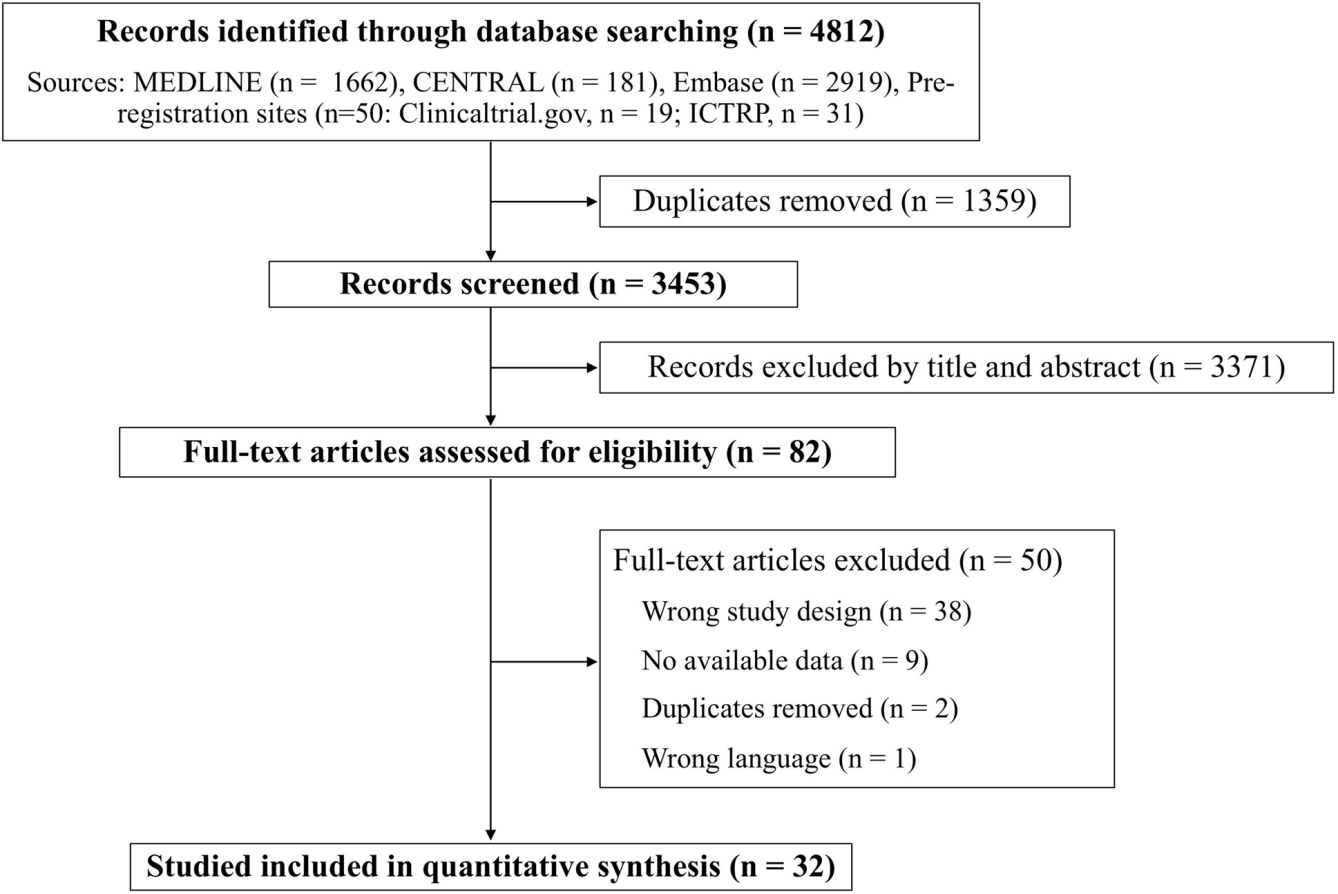
Study flow diagram. MEDLINE, Medical Literature Analysis and Retrieval System On-Line; CENTRAL, Cochrane Central Register of Controlled Trials; ICTRP, International Clinical Trials Registry Platform

The study flow diagram is shown in Fig. 1. Among the 3453 studies identified, 82 underwent full-text review after exclusion of studies that were non-diagnostic, unrelated to TCD, or that did not meet the study’s inclusion criteria. During the full-text review, data on TP, TN, FP, or FN were unavailable for 38 studies because the study designs were inappropriate for extracting these outcomes. For nine studies, contact information was unavailable, or the authors were contacted but did not provide complete data. In addition, two studies overlapped, and one study was not published in the specified language. Finally, 32 studies involving 3594 patients were included in the meta-analysis [5, 6, 24–53].

The baseline characteristics of the eligible studies are presented in Table 1 and Table S2 (Additional file). Eighteen of the included studies were prospective cohort studies. Although the detailed definition of cerebral vasospasm varied across studies, 23 studies defined it as vasospasm detectable by angiography. For the index test, the most commonly assessed MFV threshold was 120 cm/s (12 studies). For the Lindegaard ratio, the most frequently evaluated thresholds were 3 (4 studies) and 6 (4 studies). As noted above, most reference standards were performed using angiography. Seventeen studies clearly reported that the index tests were performed on the same day. Reporting on TCD frequency, the TCD-reference interval, and performance of TCD by an experienced technician was limited to 27, 17, and 21 studies, respectively. A total of 26 studies employed a reference standard that included examinations directly diagnosing vasospasm (angiography, computed tomography angiography, magnetic resonance angiography), whereas 5 studies relied solely on assessments that diagnose ischemia resulting from vasospasm (clinical evaluation, single-photon emission computed tomography, computed tomography, computed tomography perfusion).

**Table 1:** Study characteristics

Study, year	Design, No. of patients (location)	Index test			Reference standard	
		TCD frequency	MFV	LR	Diagnosis of cerebral vasospasm	TCD–reference interval
Grolimund et al. [48]	Retrospective, 79 (Switzerland)	Unknown	120	–	Angiography	Unknown
Lindegaard et al. [40]	Prospective, 125 (Norway)	Within 24 h to 25 days	–	3, 6	Angiography	Within 1 h
Sloan et al. [29]	Retrospective, 34 (USA)	Within the period of risk for vasospasm	120	–	Angiography	Within 1 day
Lewis et al. [41]	Prospective, 40 (USA)	Daily	120	–	SPECT and Clinical	On the same day
Burch et al. [53]	Retrospective, 88 (USA)	Unknown	120	–	Angiography	Unknown
Proust et al. [34]	Prospective, 37 (France)	Daily	120	–	Angiography	On the same day
Vora et al. [25]	Prospective, 145 (Canada)	Every 1 ~ 2 days	120	–	Angiography	Within 1 day
Proust et al. [49]	Prospective, 460 (France)	Every 2 days or more frequently when required	120	–	Angiography	Unknown
Jabre et al. [45]	Prospective, 28 (USA)	Unknown	150	–	Angiography	Within 1 day
Suarez et al. [28]	Prospective, 200 (USA)	Daily	120	–	Angiography	On the same day
Mascia et al. [39]	Prospective, 33 (Canada)	Daily	100	–	Angiography	On the same day
Krejza et al. [6]	Prospective, 222 (Poland)	Within 0.5 to 12 days	94, 108	3.4, 4.4	Angiography	2 h before angiography
Naval et al. [37]	Retrospective, 50 (USA)	Daily	120, 150	3, 6	Angiography	On the same day
Lee et al. [42]	Retrospective, 93 (South Korea)	Every other day	120, 180	–	CT and Clinical	Unknown
Pham et al. [35]	Prospective, 38 (Germany)	Daily	120	–	CT and CTP	On the same day
Ionita et al. [46]	Retrospective, 55 (USA)	Every other day	160	–	Angiography	Unknown
Nakae et al. [38]	Retrospective, 142 (Japan)	Daily	125	–	CT and/or Clinical	On the same day
Wang et al. [27]	Prospective, 18 (Taiwan)	On days: 1, 3, 5, 8, 10, 12, 15, 18, 22, 25, and 29	120	–	Angiography	Unknown
Kunze et al. [43]	Prospective, 50 (Germany)	Daily	140	–	Angiography	On the same day
Rajajee et al. [33]	Prospective, 81 (USA)	Daily	140	–	Angiography or CTA	On the same day
Sebastian et al. [31]	Retrospective, 267 (Canada)	Daily	120, 150	6	Angiography	On the same day
Seidel et al. [30]	Prospective, 11 (Germany)	Every other day	Unknown	–	Unknown	Unknown
Malhotra et al. [5]	Retrospective, 211 (USA)	Daily	175, 200	5, 6	Angiography	On the same day
Pifferi et al. [50]	Prospective, 37 (Italy)	Within 3 days and 7 to 10 days, and at least once Every other day after bleeding	115, 168	–	MRI + MRA	Unknown
Connolly et al. [52]	Prospective, 204 (Germany)	Unknown	120	–	Angiography	Unknown
Harst et al. [47]	Prospective, 59 (Netherlands)	On day 5 and 10	Unknown	–	CT and/or Clinical	Unknown
Wang et al. [26]	Retrospective, 105 (Chaina)	Before operation, 1 day, 2–4 days, 5–7 days, and 8–14 days after operation	120	–	Angiography	Unknown
Neulen et al. [36]	Prospective, 45 (Germany)	Twice Daily	120	–	CTA and CTP	Unknown
Sastry et al. [32]	Retrospective, 238 (USA)	Unknown	–	3	Angiography	Unknown
Clare et al. [24]	Retrospective, 33 (USA)	Daily or every other day	120	–	CTA	Unknown
Darsaut et al. [51]	Retrospective, 221 (Canada)	Daily or every other day	120	–	Angiography	Unknown
Kim et al. [44]	Retrospective, 145 (USA)	Daily	120, 150	3, 4.5	Angiography	On the same day

TCD, Transcranial Doppler; MFV, mean flow velocity; LR, Lindegaard ratio; USA, United States of America; SPECT, single-photon emission computed tomography; CT, computed tomography; CTP, computed tomography perfusion; CTA, computed tomography angiography; MRI, magnetic resonance imaging; MRA, magnetic resonance angiography

The results of the QUADAS-2 assessment are shown in Table 2. One study was judged to have a high risk of bias in the patient selection domain because inclusion were not conducted consecutively or randomly. In two studies, the TCD threshold was not pre-specified, which could affect interpretation; therefore, these studies were judged to have a high risk of bias in the index test domain. In the reference standard domain, one study was judged to have a high risk of bias because the reference test was performed with knowledge of the TCD results, which may have influenced its interpretation. In two studies, the interval between the index test and the reference standard was prolonged, making it likely that treatment occurred between tests. Therefore, a high risk of bias was determined in the flow and timing section. Additionally, the risk of bias with respect to applicability was low.

**Table 2:** QUADAS-2 results

Study	Risk of bias				Applicability concerns		
	Patient selection	Index test	Reference standard	Flow and timing	Patient selection	Index test	Reference standard
Grolimund et al. [48]	Low	Unclear	Unclear	Low	Low	Low	Low
Sloan et al. [29]	Low	Low	Low	Low	Low	Low	Low
Lindegaard et al. [40]	Low	Unclear	Unclear	Unclear	Low	Low	Low
Lewis et al. [41]	Low	Unclear	Unclear	Low	Low	Low	Low
Burch et al. [53]	Low	Low	Low	Unclear	Low	Low	Low
Vora et al. [25]	Low	Low	Low	Unclear	Low	Low	Low
Proust et al. [34]	Low	Low	Low	Low	Low	Low	Low
Proust et al. [49]	Low	Unclear	Unclear	High	Low	Low	Low
Suarez et al. [28]	Low	Unclear	Unclear	High	Low	Low	Low
Jabre et al. [45]	Low	Low	Low	Low	Low	Low	Low
Mascia et al. [39]	Low	Low	Low	Low	Low	Low	Low
Krejza et al. [6]	Low	Low	Low	Low	Low	Low	Low
Naval et al. [37]	Low	Unclear	Unclear	Unclear	Low	Low	Low
Lee et al. [42]	Low	Unclear	Low	Unclear	Low	Low	Low
Pham et al. [35]	Low	Low	Low	Low	Low	Low	Low
Ionita et al. [46]	Low	Unclear	Low	Low	Low	Low	Low
Nakae et al. [38]	Low	High	Unclear	Unclear	Low	Low	Low
Wang et al. [27]	Low	Unclear	Unclear	Unclear	Low	Low	Low
Kunze et al. [43]	High	Low	Low	Unclear	Low	Low	Low
Rajajee et al. [33]	Low	High	Unclear	Low	Low	Low	Low
Sebastian et al. [31]	Low	Unclear	High	Low	Low	Low	Low
Seidel et al. [30]	Low	Unclear	Unclear	Unclear	Low	Low	Low
Malhotra et al. [5]	Low	Low	Low	Low	Low	Low	Low
Pifferi et al. [50]	Low	Unclear	Unclear	Low	Low	Low	Low
Connolly et al. [52]	Low	Low	Low	Low	Low	Low	Low
Harst et al. [47]	Low	Unclear	Unclear	Low	Low	Low	Low
Wang et al. [26]	Low	Unclear	Unclear	Low	Low	Low	Low
Neulen et al. [36]	Low	Low	Low	Low	Low	Low	Low
Sastry et al. [32]	Low	Unclear	Unclear	Low	Low	Low	Low
Clare et al. [24]	Low	Low	Low	Unclear	Low	Low	Low
Darsaut et al. [51]	Low	Unclear	Low	Low	Low	Low	Low
Kim et al. [44]	Low	Low	Unclear	Low	Low	Low	Low

**Fig. 2:**
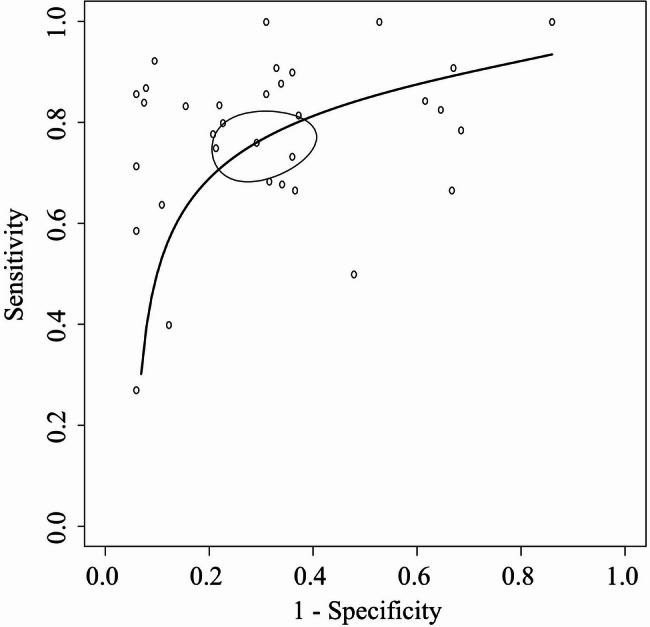
Summary receiver operating characteristic curve The summary receiver operating characteristic plots of the bivariate meta-analysis for detecting cerebral vasospasm by transcranial Doppler. The plots show sensitivity and 1-specificity of individual studies on the diagnostic accuracy of transcranial Doppler. TCD thresholds followed the settings in each study. The ellipse around the point estimate represents a 95% CI. The ROC curve is restricted to the range of specificities for each study

The SROC curves for detecting cerebral vasospasm using TCD are shown in Fig. 2, and summary estimates of sensitivity, specificity, AUC, positive likelihood ratio, and negative likelihood ratio are presented in Table 3. In Fig. 2, the solid curves are the SROC curves integrated into a bivariate random-effects model for diagnosing cerebral vasospasm by TCD. The dots represent point estimates of sensitivity and 1-specificity for each included study, and the ellipses represent 95% CIs for sensitivity and 1-specificity. In the primary meta-analysis, when diagnostic accuracy was evaluated using both MFV and the Lindegaard ratio, preference was given to diagnostic accuracy based on MFV. When multiple thresholds were reported, analyses incorporated diagnostic accuracy using an MFV threshold of 120 cm/s (or the closest available value) and a Lindegaard ratio threshold of 3 (or the closest available value). The AUC of the SROC curve for diagnosing cerebral vasospasm using TCD was approximately 0.82. The pooled sensitivity and specificity were 76% (95% CI: 70–81%) and 77% (68–84%), respectively. The positive likelihood ratio was 3.4, and the negative likelihood ratio was 0.3. The *I*^2^ value, a measure of heterogeneity, was 12.0%. Forest plots for each study are shown in Figure S1 and S2.

**Table 3:** Sensitivities, specificities, AUROCs, and likelihood ratios by subgroups

Subgroups	No. of Patients (study)	Sensitivity	Specificity	AUROC	Positive likelihood ratio	Negative likelihood ratio
**All** ^**a, b**^	**3594 (32)**	**0.76 (0.70–0.81)**	**0.77 (0.68–0.84)**	**0.82 (0.74–0.83)**	**3.4 (2.8–4.7)**	**0.32 (0.24–0.40)**
MFV threshold-based ^a, c^	3161 (28)	0.76 (0.69–0.81)	0.75 (0.66–0.83)	0.81 (0.74–0.82)	3.0 (2.3–4.3)	0.33 (0.26–0.40)
LR threshold-based ^a, d^	1162 (7)	0.82 (0.58–0.94)	0.88 (0.64–0.97)	0.91 (0.77–0.95)	6.4 (2.5–20.0)	0.21 (0.08–0.46)
Low ROB ^a^	1223 (9)	0.79 (0.72–0.85)	0.82 (0.66–0.91)	0.84 (0.74–0.90)	4.7 (2.2–9.3)	0.26 (0.17–0.39)

AUROC, area under the receiver operating characteristic curve; MFV, mean flow velocity; LR, Lindegaard ratio; ROB, risk of bias

^a^ Heterogeneity (subgroup, *I*^2^%): All, 12%; MFV threshold-based 11%: LR threshold-based, 57%; Low ROB, 25%

^b^ If both MFV and LR were reported, MFV-based accuracy was extracted. Thresholds were 120 cm/s for MFV and 3 for LR (or the closest reported thresholds)

^c^ When multiple MFV thresholds were evaluated, diagnostic accuracy was extracted at 120 cm/s (or the closest available threshold)

^d^ When multiple LR thresholds were evaluated, diagnostic accuracy was extracted at 3 (or the closest available threshold)

The results of subgroup analyses across different thresholds are presented in Table 3 and Table S3. Meta-analyses were performed using studies that evaluated MFV with a threshold of 120 cm/s (or the closest available value) and studies that evaluated the Lindegaard ratio with a threshold of 3 (or the closest available value); these results are shown in Table 3. Additional meta-analyses were performed for MFV at 120 cm/s, MFV thresholds exceeding 120 cm/s, Lindegaard ratio at 3, and Lindegaard ratio at 6, with findings summarized in Table 3 and Table S3. Because MFV thresholds exceeding 120 cm/s did not share a common cutoff, a pooled meta-analysis was performed including all studies that applied thresholds above 120 cm/s. In subgroups with MFV thresholds exceeding 120 cm/s and with a higher Lindegaard ratio threshold of 6 (vs. 3), specificity and the positive likelihood ratio increased, whereas sensitivity decreased and the negative likelihood ratio increased. This pattern was more pronounced for the Lindegaard ratio. Studies that used the Lindegaard ratio showed meta-analysis results with higher diagnostic accuracy than those of the main analysis; however, the number of studies included in these meta-analyses was small. Subgroup analyses by aSAH severity and by diagnostic criteria in the reference standard were initially planned; however, they were not conducted because sufficient valid data for appropriate classification were unavailable.

Sensitivity analyses were performed using meta-analyses for the following subgroups: studies assessed as having a low risk of bias, prospective studies, and studies providing clear information on TCD frequency, the TCD–reference interval, performance of TCD by an experienced technician, and studies employing a reference standard with angiography or without angiography. The results are presented in Table 3 and Table S4. Studies at low risk of bias demonstrated slightly higher diagnostic accuracy than that observed in the primary analysis. Studies employing a reference standard without angiography demonstrated lower diagnostic accuracy compared to other subgroups. Findings from the other subgroup analyses were broadly consistent with those of the primary meta-analysis.

## Discussion

In this systematic review and meta-analysis, we evaluated the diagnostic accuracy of TCD ultrasonography for detecting MCA vasospasm after aSAH. The pooled estimates demonstrated a sensitivity of 76%, a specificity of 77%, a positive likelihood ratio of 3.4, and a negative likelihood ratio of 0.3 for detecting MCA vasospasm using either mean flow velocity or the Lindegaard ratio. In subgroup analyses, the Lindegaard ratio showed superior diagnostic accuracy compared with the absolute value of MFV.

Prior meta-analyses investigating TCD for diagnosing cerebral vasospasm and DCI reported sensitivities ranging from 57% to 90%, specificities from 68% to 75%, positive predictive values from 32% to 58%, negative predictive values from 90% to 92%, positive likelihood ratios between 1.8 and 3.4, and negative likelihood ratios between 0.1 and 0.6 [11–13]. These findings are consistent with our results. In our study, the observed positive likelihood ratio of 3.4 and negative likelihood ratio of 0.3 suggest that TCD can meaningfully shift pre-test to post-test probability and support its use as an adjunctive point-of-care tool in the management of patients with aSAH. However, assuming a general prevalence of cerebral ischemia of 30% [54], the likelihood ratios derived in this study yield post-test probabilities of 59% for a positive result and 11% for a negative result. These findings indicate that a positive TCD result alone is unlikely to confirm the diagnosis with certainty, and a negative result alone is insufficient to definitively rule it out. Furthermore, compared with previous studies, our results showed lower sensitivity, suggesting that caution is warranted when using TCD as a rule-out tool. This finding may reflect the fact that our systematic review focused on studies assessing the MCA [55]. Comprehensive monitoring of all visualizable vessels, along with integration with other modalities, remain crucial for accurate evaluation [4, 56].

Previous systematic reviews have evaluated the diagnostic accuracy of TCD across all cerebral arteries, including the posterior circulation, rather than focusing solely on the MCA [11–13]. In contrast, this study is the first meta-analysis to specifically focus on the MCA, which is the most commonly monitored vessel in the clinical management of aSAH. Given that optimal cutoff values in TCD vary by vessel and that studies investigating vessels other than the MCA remain scarce [57–59], integrating diagnostic accuracy across multiple vascular territories may be inappropriate. Focusing on the MCA allow the study to provide findings that are both clinically meaningful and readily generalizable in SAH management.

Subgroup analyses revealed that the Lindegaard ratio demonstrated superior diagnostic performance compared with the absolute value of MFV. The Lindegaard ratio is a TCD index calculated as the MFV for the MCA divided by that in the ipsilateral extracranial internal carotid artery [17]. It is useful for distinguishing true cerebral vasospasm from hyperemia caused by increased cerebral blood flow [17]. This finding aligns with previous systematic reviews and reinforces the utility of the Lindegaard ratio [13]. Previous studies have also reported that temporal increases in the MFV for the MCA may aid in the diagnosis of cerebral vasospasm and DCI in addition to assessment based on a single TCD measurement [60, 61]. Therefore, in clinical practice, it is reasonable not to rely solely on absolute flow-velocity values but also to assess other bedside-obtainable indices. However, evidence supporting Lindegaard ratio–based thresholds in this meta-analysis was limited, given the small number of included studies, and the pooled estimates were imprecise with confidence intervals; accordingly, findings based on the Lindegaard ratio should be interpreted with caution. Future studies are warranted to validate these approaches.

Concerning research perspectives in this field, our study identified several methodological heterogeneities. As shown in Table 1, the timing and frequency of TCD examinations, the definition of cerebral vasospasm, and the interval between TCD and the reference standard varied across studies. Despite some variation in diagnostic methods, all included studies evaluated the same pathological condition—vasospasm associated with SAH—which likely contributed to the similar diagnostic accuracy observed across studies and, consequently, to the relatively low statistical heterogeneity. However, this statistical consistency does not necessarily imply uniform clinical applicability; rather, it remains uncertain to which specific clinical scenarios the diagnostic accuracy estimates from this meta-analysis can be generalized. Additionally, among the 32 included studies, 14 (approximately half) were retrospective in design, and each QUADAS-2 risk-of-bias domain contained one or two items rated as high risk. Future research should prioritize prospective studies with rigorously defined index tests and reference standards to reduce methodological heterogeneity. In addition, interventional studies assessing the clinical impact of incorporating TCD-based protocols into standard aSAH management—particularly whether early detection and treatment based on TCD findings improves functional outcomes—are needed to establish the utility of TCD beyond diagnostic accuracy metrics.

A key strength of this review is that it represents the most up-to-date systematic evaluation of the diagnostic accuracy of TCD for detecting cerebral vasospasm following aSAH. Incorporating recent studies and re-evaluating the evidence allowed us to include a larger number of studies and patients (32 studies, 3,594 patients) compared with previous systematic reviews, which enabled a more precise estimation of diagnostic accuracy with narrower CIs. However, this study has several limitations. First, the diagnosis of cerebral vasospasm was based on study-specific definitions. Although a universally accepted definition of DCI has not yet been established, recent consensus statements have proposed that DCI be defined by the following criteria: (1) a decrease of 2 or more points in the Glasgow Coma Scale or the National Institutes of Health Stroke Scale; (2) the appearance of new neurological abnormalities persisting for more than 1 h; and (3) the absence of alternative explanations such as rebleeding, hydrocephalus, infection, or metabolic disturbances [54]. It has been increasingly recognized that vasospasm is not the sole cause of DCI [56, 62–64]. However, few studies included in this review applied this consensus-based definition, which may limit the generalizability of our findings to contemporary clinical practice. Furthermore, our subgroup analysis demonstrated that the diagnostic accuracy of TCD was low in the subgroup where angiography was excluded as the reference standard-namely, the subgroup focused on diagnosing ischemia following vasospasm. These findings suggest that while TCD directly detects cerebral vasospasm, it does not necessarily identify the resulting ischemia. In future research, it may be appropriate to adopt a standardized definition of DCI based on clinical diagnostic criteria, without necessarily requiring confirmation of anatomical vasospasm findings. Second, the time interval between TCD and the reference standard was not standardized. In clinical practice, when an elevated MFV is detected on TCD or when neurological deterioration suggests cerebral vasospasm, therapeutic interventions are likely initiated. Consequently, treatments may be performed before angiography, potentially alleviating vasospasm and resulting in an underestimation of diagnostic accuracy. Third, it was unclear in 14 of the 32 included studies whether the reference standard assessment was blinded to TCD results. In some studies, the same clinician performed both TCD examinations and DCI diagnosis, introducing a potential risk of overestimated diagnostic accuracy due to observer bias. Nevertheless, in clinical practice, a positive TCD result alone is unlikely to prompt immediate invasive treatment; instead, confirmatory testing would typically follow to establish a more definitive diagnosis. Greater caution is warranted in cases of false negatives, and when clinical suspicion persists, alternative diagnostic approaches should be pursued without hesitation. Finally, the optimal TCD threshold for clinical decision-making could not be determined. MFV of 120 cm/s and Lindegaard ratio of 3 were the most frequently evaluated thresholds, and subgroup meta-analyses indicated that increasing either threshold tended to show higher specificity and lower sensitivity. Accordingly, thresholds of approximately 120 cm/s for MFV and 3 for the Lindegaard ratio may be reasonable. However, this interpretation was not based on a systematic evaluation across multiple threshold values. Future prospective studies systematically comparing standardized cutoff values are warranted.

## Conclusions

TCD demonstrates promising diagnostic accuracy for detecting cerebral vasospasm. Incorporating TCD into multimodal neuromonitoring may improve the management of aSAH. However, studies applying the recent standardized definition of DCI or evaluating the diagnostic performance of emerging TCD assessment methods, such as the Lindegaard ratio, remain limited, highlighting the need for further research.

## Supplementary Information

Supplementary Material 1

## Data Availability

Data supporting the findings of this study are available from the corresponding author upon reasonable request.

## Abbreviations

aSAH: Aneurysmal subarachnoid hemorrhage
DCI: Delayed cerebral ischemia
TCD: Transcranial Doppler
POCUS: Point-of-care ultrasound
MCA: Middle cerebral artery
PRISMA-DTA: Preferred Reporting Items for Systematic Review and Meta-analysis of Diagnostic Test Accuracy Studies
MFV: Mean blood flow velocity
MEDLINE: Medical Literature Analysis and Retrieval System Online
CENTRAL: Cochrane Central Register of Controlled Trials
ICTRP: International Clinical Trials Registry Platform
TP: True-positive
FP: False-positive
TN: True-negative
FN: False-negative
QUADAS-2: Quality Assessment of Diagnostic Accuracy Studies 2 tool
Cis: Confidence intervals
SROC: Summary receiver operating characteristic
AUCs: Areas under the curves
