# Supplementary material for: Diagnostic accuracy of transcranial Doppler for cerebral vasospasm in aneurysmal subarachnoid hemorrhage: a systematic review and meta-analysis
Source: Crit Care. 2026 Feb 2;30:101. doi: 10.1186/s13054-026-05849-6 (PMC12952058; doi:10.1186/s13054-026-05849-6)

**Table S1:** **Full Search Strategy**

| Database | Search term |
| --- | --- |
| MEDLINE | |
| 1 | "Subarachnoid Hemorrhage"[MeSH Terms] OR ("intracranial hemorrhages"[MeSH Terms:noexp] OR "Cerebral Hemorrhage"[MeSH Terms:noexp] OR "Hemorrhagic Stroke"[MeSH Terms]) OR ("Subarachnoid Hemorrhage"[Title/Abstract:~6] OR "Subarachnoid Hemorrhages"[Title/Abstract:~6] OR "Subarachnoid Haemorrhage"[Title/Abstract:~6] OR "Subarachnoid Haemorrhages"[Title/Abstract:~6] OR "Subarachnoid bleeding"[Title/Abstract:~6] OR "Subarachnoid Bleedings"[Title/Abstract:~6]) OR "cerebral aneurysm*"[Title/Abstract] OR "intracranial aneurysm*"[Title/Abstract] OR ("intracranial aneurysm"[MeSH Terms] AND "rupture, spontaneous"[MeSH Terms]) OR ("aneurysm, ruptured"[MeSH Terms:noexp] AND ("brain"[MeSH Terms] OR "Meninges"[MeSH Terms])) |
| 2 | "ultrasonography, doppler, transcranial"[MeSH Terms] |
| 3 | neurosono*[TW] |
| 4 | echoencephalograph*[TW] |
| 5 | echo-encephalograph*[TW] |
| 6 | ultraso*[TW] |
| 7 | sonograph*[TW] |
| 8 | echograph*[TW] |
| 9 | "doppler*"[TW] |
| 10 | duplex[TW] |
| 11 | "color flow*"[tiab] OR "colour flow*"[tiab] OR "color coded"[TW] OR "colour coded"[TW] |
| 12 | #2 OR #3 OR #4 OR #5 OR #6 OR #7 OR #8 OR #9 OR #10 OR #11 |
| 13 | "transcranial"[TW] OR trans-cranial[TW] |
| 14 | neurovascular[TW] |
| 15 | #13 OR #14 |
| 16 | #12 AND #15 |
| 17 | "TCD"[tiab] OR "TCCS"[tiab] |
| 18 | #16 OR #17 |
| 19 | #1 AND #18 |
|  |  |
| CENTRAL | |
| 1 | MeSH descriptor: [Subarachnoid Hemorrhage] explode all trees |
| 2 | (subarachnoid NEAR h?emorrhage*):ti,ab,kw |
| 3 | (subarachnoid NEAR bleeding*):ti,ab,kw |
| 4 | #1 #2 OR #3 |
| 5 | (cerebral aneurysm*):ti,ab,kw |
| 6 | (intracranial aneurysm*):ti,ab,kw |
| 7 | MeSH descriptor: [Intracranial Aneurysm] explode all trees |
| 8 | MeSH descriptor: [Rupture, Spontaneous] explode all trees |
| 9 | #7 AND #8 |
| 10 | MeSH descriptor: [Aneurysm, Ruptured] this term only |
| 11 | MeSH descriptor: [Brain] explode all trees |
| 12 | MeSH descriptor: [Meninges] explode all trees |
| 13 | #11 or #12 |
| 14 | #10 and #13 |
| 15 | #4 or #5 or #6 or #9 or #14 |
| 16 | MeSH descriptor: [Ultrasonography, Doppler, Transcranial] explode all trees |
| 17 | (neurosono* or echoencephalograph* or echo-encephalograph* or ultraso* or sonograph* or echograph* or doppler* or duplex):ti,ab,kw |
| 18 | ((color or colour) NEAR/2 (flow* or coded)):ti,ab,kw |
| 19 | #16 or #17 or #18 |
| 20 | #15 and #19 |
| 21 | #20 in Trials |
|  |  |
| Embase | |
| 1 | 'subarachnoid hemorrhage'/de |
| 2 | subarachnoid NEAR/6 h?emorrhage* |
| 3 | subarachnoid NEAR/6 bleeding* |
| 4 | #1 OR #2 OR #3 |
| 5 | 'intracranial aneurysm'/exp |
| 6 | 'rupture'/de |
| 7 | #5 AND #6 |
| 8 | 'aneurysm rupture'/de |
| 9 | 'brain'/exp |
| 10 | 'meninx'/exp |
| 11 | #9 OR #10 |
| 12 | #8 AND #11 |
| 13 | #4 OR #12 |
| 14 | 'transcranial doppler ultrasonography'/exp |
| 15 | neurosono*:ti,ab,kw OR echoencephalograph*:ti,ab,kw OR 'echo encephalograph*':ti,ab,kw OR ultraso*:ti,ab,kw OR sonograph*:ti,ab,kw OR echograph*:ti,ab,kw OR doppler*:ti,ab,kw OR duplex:ti,ab,kw |
| 16 | ((color OR colour) NEAR/2 (flow* OR coded)):ti,ab,kw |
| 17 | #14 OR #15 OR #16 |
| 18 | #13 AND #17 |
| 19 | 'subarachnoid hemorrhage'/de |
| 20 | subarachnoid NEAR/6 h?emorrhage* |
| 21 | subarachnoid NEAR/6 bleeding* |
| 22 | #19 OR #20 OR #21 |
| 23 | 'intracranial aneurysm'/exp |
| 24 | 'rupture'/de |
| 25 | #23 AND #24 |
| 26 | 'aneurysm rupture'/de |
| 27 | 'brain'/exp |
| 28 | 'meninx'/exp |
| 29 | #27 OR #28 |
| 30 | #26 AND #29 |
| 31 | #22 OR #30 |
| 32 | 'transcranial doppler ultrasonography'/exp |
| 33 | neurosono*:ti,ab,kw OR echoencephalograph*:ti,ab,kw OR 'echo encephalograph*':ti,ab,kw OR ultraso*:ti,ab,kw OR sonograph*:ti,ab,kw OR echograph*:ti,ab,kw OR doppler*:ti,ab,kw OR duplex:ti,ab,kw |
| 34 | ((color OR colour) NEAR/2 (flow* OR coded)):ti,ab,kw |
| 35 | #32 OR #33 OR #34 |
| 36 | #31 AND #35 |
|  |  |

**Table S2:** **Additional study characteristics.**

| Study, year | Conflict of interest | Funding source | Severity of SAH | | | | Index test | | Reference  standard |
| --- | --- | --- | --- | --- | --- | --- | --- | --- | --- |
|  |  |  | WFNS (median) | Hunt & Hess (median) | Fisher (median) | mFisher (median) | TCD by experienced technician | Reference blinded | TCD blinded |
| Grolimund et al, 1987 | Unknown | Unknown | Unknown | Unknown | Unknown | Unknown | Unknown | Unknown | Unknown |
| Lindegaard et al, 1989 | Unknown | Yes^1^ | Unknown | Unknown | Unknown | Unknown | Unknown | Unknown | Unknown |
| Sloan et al, 1989 | Unknown | Unknown | Unknown | Unknown | Unknown | Unknown | Yes | Yes | Yes |
| Lewis et al, 1992 | Unknown | Unknown | Unknown | Unknown | Unknown | Unknown | Yes | Unknown | Yes |
| Burch et al, 1996 | Unknown | Unknown | Unknown | Unknown | Unknown | Unknown | Unknown | Yes | Yes |
| Proust et al, 1999 | Unknown | Unknown | Unknown | 3 | Unknown | Unknown | Yes | Yes | Yes |
| Vora et al, 1999 | Unknown | Unknown | 2 | Unknown | Unknown | Unknown | Unknown | Yes | Yes |
| Proust et al, 2002 | Unknown | Unknown | Unknown | 3 | 3 | Unknown | Unknown | Unknown | Unknown |
| Jabre et al, 2002 | Unknown | Unknown | Unknown | Unknown | Unknown | Unknown | Yes | Yes | Yes |
| Suarez et al, 2002 | Unknown | Unknown | Unknown | Unknown | 3 | Unknown | Yes | Unknown | Unknown |
| Mascia et al, 2003 | Yes^2^ | Yes^3^ | 2.5 | Unknown | 3 | Unknown | Yes | Yes | Yes |
| Krejza et al, 2005 | No | Yes^4^ | 1 | Unknown | Unknown | Unknown | Yes | Yes | Yes |
| Naval et al, 2005 | Unknown | Unknown | Unknown | 3 | Unknown | Unknown | Yes | Unknown | Unknown |
| Lee et al, 2006 | Unknown | Unknown | 3 | Unknown | 3 | Unknown | Yes | Unknown | Yes |
| Pham et al, 2007 | Unknown | Unknown | Unknown | Unknown | 4 | Unknown | Yes | Yes | Yes |
| Ionita et al, 2008 | Unknown | Unknown | Unknown | Unknown | Unknown | Unknown | Yes | Unknown | Yes |
| Nakae et al, 2011 | No | Unknown | Unknown | Unknown | Unknown | 2.5 | Yes | Unknown | Unknown |
| Wang et al, 2012 | Unknown | Unknown | Unknown | Unknown | Unknown | Unknown | Yes | Unknown | Unknown |
| Kunze et al, 2012 | Unknown | Yes^5^ | Unknown | Unknown | Unknown | Unknown | Yes | Yes | Yes |
| Rajajee et al, 2012 | No | Unknown | Unknown | 2.5 | Unknown | 3 | Yes | Yes | Unknown |
| Sebastian et al, 2013 | Unknown | Unknown | Unknown | 2 | Unknown | Unknown | Yes | Unknown | no |
| Seidel et al, 2014 | Unknown | Unknown | 2.5 | 1.5 | Unknown | Unknown | Unknown | Unknown | Unknown |
| Malhotra et al, 2014 | Unknown | Unknown | Unknown | 3 | 3 | Unknown | Yes | Yes | Yes |
| Pifferi et al, 2014 | Unknown | Unknown | Unknown | Unknown | Unknown | Unknown | Unknown | Unknown | Unknown |
| Connolly et al, 2017 | No | Unknown | Unknown | Unknown | Unknown | Unknown | Yes | Yes | Yes |
| Harst et al, 2017 | Unknown | Unknown | Unknown | Unknown | Unknown | Unknown | Unknown | Unknown | Unknown |
| Wang et al, 2018 | No | No | Unknown | 3 | Unknown | Unknown | Unknown | Unknown | Unknown |
| Neulen et al, 2019 | No | Yes^6^ | Unknown | Unknown | Unknown | Unknown | Yes | Yes | Yes |
| Sastry et al, 2022 | No | Unknown | Unknown | Unknown | Unknown | Unknown | Unknown | Unknown | Unknown |
| Clare et al, 2022 | Yes^7^ | No | Unknown | 3 | Unknown | Unknown | robotic | Yes | Yes |
| Darsaut et al, 2022 | Unknown | Unknown | Unknown | Unknown | Unknown | Unknown | Yes | Unknown | Yes |
| Kim et al, 2023 | No | No | Unknown | 3 | Unknown | 3 | Unknown | Yes | Unknown |

Conflict of interest and Funding source

^1^ The Norwegian Council on Cardiovascular Diseases.

^2^ Doppler equipment supplied by DWL, Elektronische Systeme, Supplingen, Germanny.

^3^ Foundation Baxter and Alma Ricard Chair in cerebrovascular surgery, University of Toronto.

^4^ Supported in part by the American Heart Association Established Investigator Award and NATO fellowship program.

^5^ The German Research Foundation (DFG) and the University of Wuerzburg under the "Open Access Publishing" program.

^6^ A grant of the Medical Center of the Johannes Gutenberg University Mainz (Stufe I Foerderung, grant to A.N.).

^7^ One author was the Co-Founder & Chief Scientific Officer of NovaSignal.

**Table S3:** **Sensitivities, specificities, AUROCs, and likelihood ratios by threshold-based subgroups.**

| Subgroups | No. of Patients (study) | Sensitivity | Specificity | AUROC | Positive likelihood ratio | Negative likelihood ratio |
| --- | --- | --- | --- | --- | --- | --- |
| MFV threshold = 120 cm/s ^a^ | 2302 (19) | 0.75 (0.69 - 0.81) | 0.74 (0.63 - 0.83) | 0.80 (0.73 - 0.82) | 2.9 (2.1 - 4.1) | 0.34 (0.27 - 0.41) |
| MFV threshold > 120 cm/s ^a, b^ | 1137 (11) | 0.70 (0.56 - 0.81) | 0.81 (0.66 - 0.90) | 0.81 (0.74 - 0.83) | 3.7 (2.3 - 6.1) | 0.38 (0.27 - 0.50) |
| LR threshold = 3 ^a^ | 558 (4) | 0.91 (0.85 - 0.95) | 0.83 (0.36 - 0.98) | 0.92 (0.86 - 0.95) | 5.3 (1.5 - 37.5) | 0.12 (0.08 - 0.21) |
| LR threshold = 6 ^a^ | 557 (4) | 0.34 (0.21 - 0.49) | 0.97 (0.91 - 0.99) | 0.73 (0.39 - 0.96) | 11.7 (4.9 - 29.0) | 0.68 (0.54 - 0.80) |

AUROC, area under the receiver operating characteristic curve; MFV, mean flow velocity; LR, Lindegaard ratio.

^a^ Heterogeneity (subgroup, *I*^2^ %): 1, 12%;2, 25%: 3, 0%; 4, 0%.

^b^ Varied across 11 assessments (125–200 cm/s): 125 (n=1), 140 (n=2), 150 (n=4), 160 (n=1), 168 (n=1), 180 (n=1), and 200 cm/s (n=1).

**Table S4:** **Sensitivities, specificities, AUROCs, and likelihood ratios by study-design subgroups.**

| Subgroups | No. of Patients (study) | Sensitivity | Specificity | AUROC | Positive likelihood ratio | Negative likelihood ratio |
| --- | --- | --- | --- | --- | --- | --- |
| Prospective studies ^a^ | 1833 (18) | 0.77 (0.71 - 0.83) | 0.76 (0.62 - 0.86) | 0.82 (0.71 - 0.84) | 3.3 (2.0 - 5.7) | 0.30 (0.22 - 0.41) |
| TCD frequency ^a, b^ | 2957 (27) | 0.76 (0.69 - 0.81) | 0.75 (0.66 - 0.83) | 0.81 (0.72 - 0.82) | 3.0 (2.2 - 4.3) | 0.33 (0.25 - 0.42) |
| TCD-reference gap ^a, b^ | 1848 (17) | 0.78 (0.72 - 0.83) | 0.73 (0.59 - 0.84) | 0.81 (0.72 - 0.83) | 2.9 (1.9 - 4.8) | 0.30 (0.22 - 0.42) |
| TCD experienced technician ^a, b^ | 2102 (21) | 0.76 (0.69 - 0.82) | 0.73 (0.60 - 0.83) | 0.80 (0.71 - 0.82) | 2.8 (1.9 - 4.2) | 0.33 (0.25 - 0.43) |
| Diagnosis with angiography ^a, c^ | 3211 (26) | 0.78 (0.71 - 0.84) | 0.81 (0.72 - 0.88) | 0.86 (0.78 - 0.87) | 4.2 (2.9 - 6.3) | 0.27 (0.21 - 0.35) |
| Diagnosis without angiography ^a, c^ | 372 (5) | 0.70 (0.59 - 0.80) | 0.51 (0.37 - 0.65) | 0.68 (0.52 - 0.76) | 1.4 (1.1 - 2.0) | 0.58 (0.40 - 0.82) |

AUROC, area under the receiver operating characteristic curve. TCD, transcranial Doppler.

a Heterogeneity (subgroup, I2 %): Prospective studies, 32%; TCD frequency, 13%: TCD-reference interval, 18%; TCD experienced technician, 15%; Diagnosis with angiography, 14%; Diagnosis without angiography, 0%.

^b^ Only studies with available data

^c^ Studies employing a reference standard with angiography or without angiography

**Figure S1: Forrest plot for sensitivity**


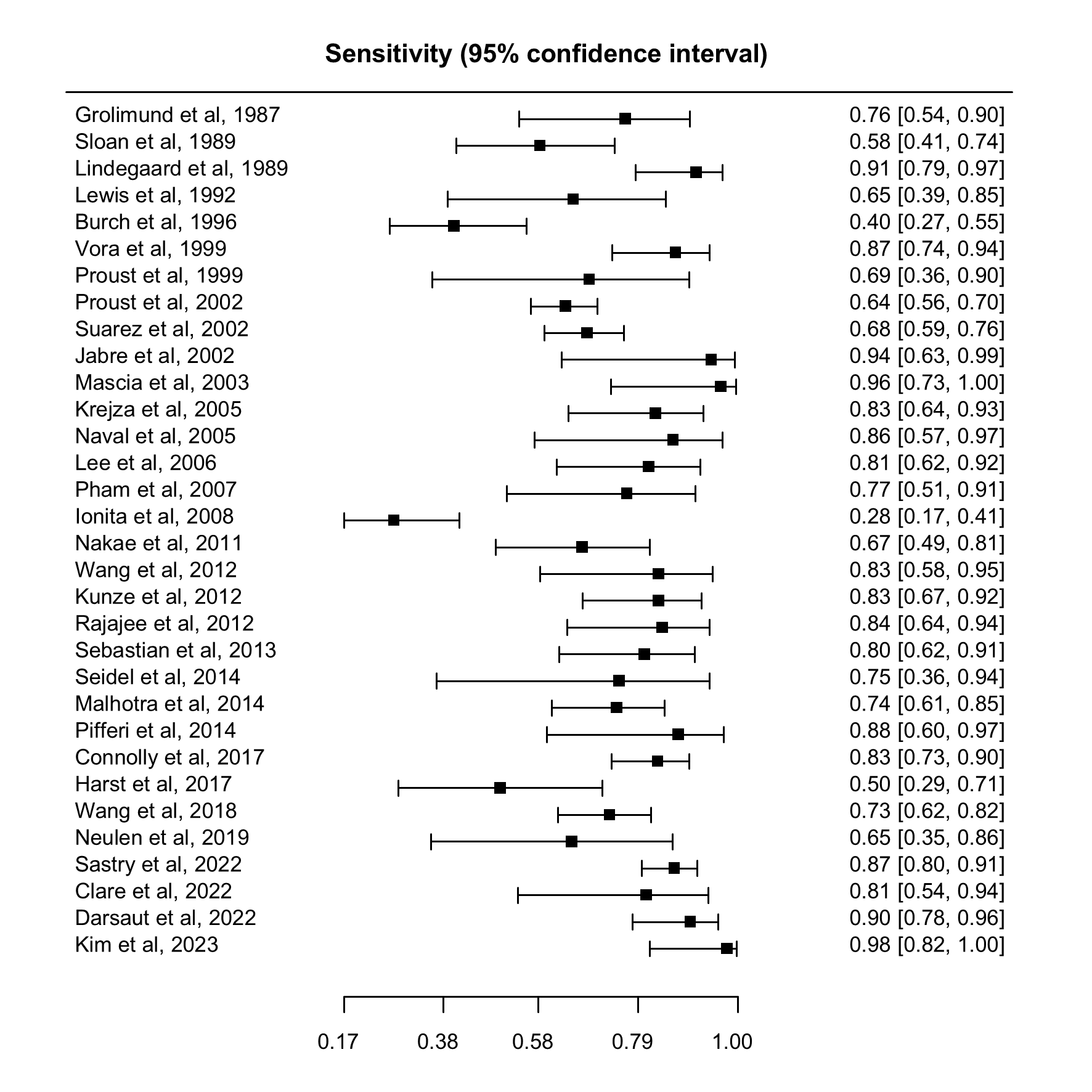


**Figure S2: Forrest plot for specificity**


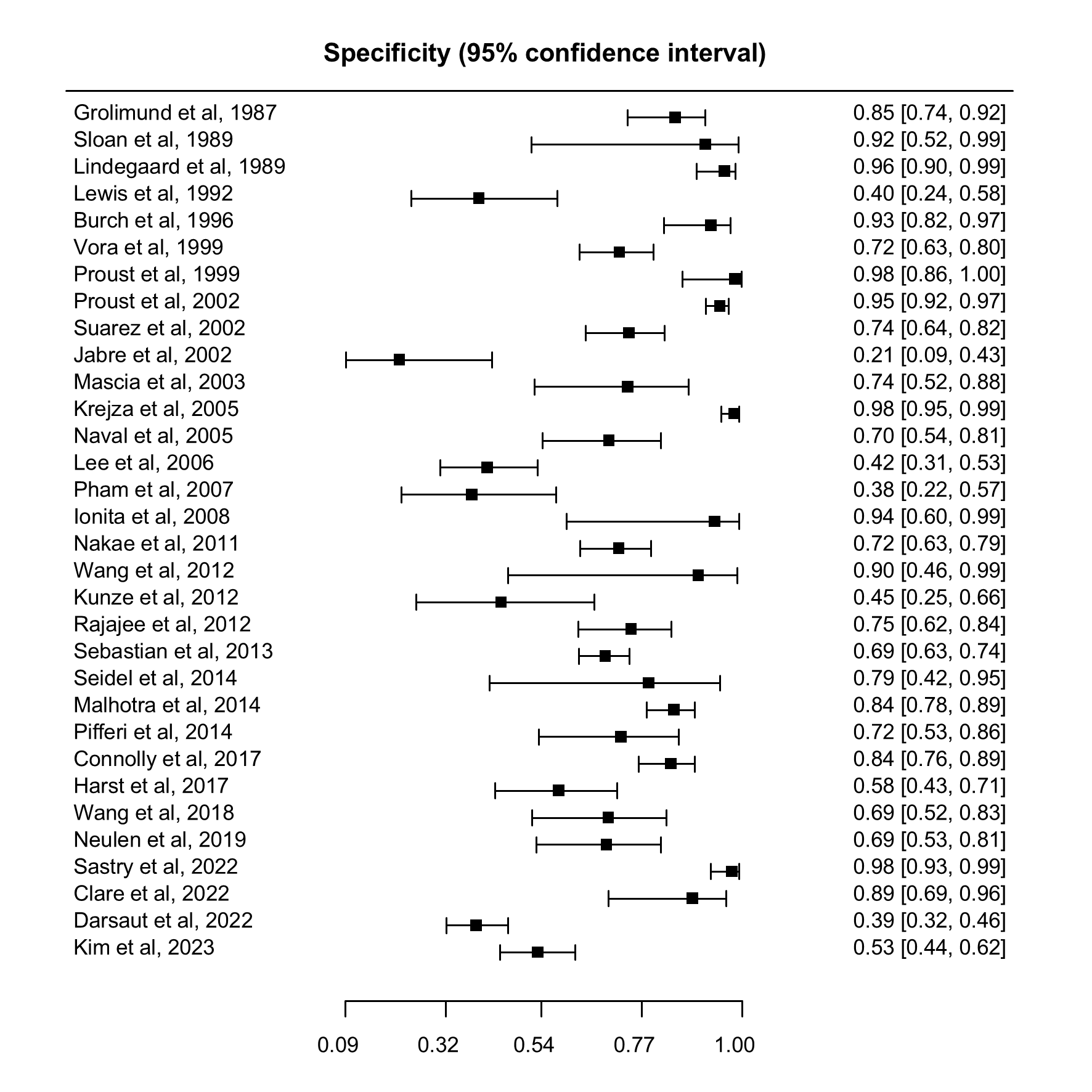

Supplement: Supplementary file 1 — Supplementary Material 1 [file 13054_2026_5849_MOESM1_ESM.docx]
